# Effects of PMMA spacer loaded with varying vancomycin concentrations on bone regeneration in the Masquelet technique

**DOI:** 10.1038/s41598-022-08381-z

**Published:** 2022-03-11

**Authors:** Jie Xie, Wu Wang, Xiaolei Fan, Hui Li, Haoyi Wang, Runzhi Liao, Yihe Hu, Min Zeng

**Affiliations:** 1 Department of Orthopedics, Xiangya Hospital, Central South University, No. 87 Xiangya Road, Changsha, 410008 Hunan China; 2 National Clinical Research Center for Geriatric Disorders, Xiangya Hospital, Central South University, No. 87 Xiangya Road, Changsha, 410008 Hunan China; 3 Department of Orthopedics, The Fifth Affiliated Hospital of Xinjiang Medical University, Urumqi, 830011 Xinjiang China

**Keywords:** Trauma, Experimental models of disease, Preclinical research

## Abstract

Whether antibiotics should be included remains greatly debated in Masquelet technique. This study intended to determine the effect of polymethyl methacrylate (PMMA) spacer loaded with different vancomycin concentrations on bone defect repair. Hollow cylindrical spacers consisting of PMMA and varying vancomycin concentrations (0, 1, 2, 4, 6, 8, and 10 g) were prepared. Critical bone defects of rabbits were created at the radial shaft, and spacers were implanted and subsequently intramedullary fixed with retrograde Kirschner’s wires (n = 4 for each vancomycin concentration). After 4 weeks, the induced membranes were opened and cancellous allografts were implanted into the defects. Eight weeks post-operatively, the results of X-ray, histology, and micro-CT revealed that some cortical bone was formed to bridge the gap and the bone marrow cavity was formed over time. Quantitatively, there was more new bone formation in the groups with a relatively lower vancomycin concentration (1–4 g) compared with that in the groups with a higher vancomycin concentration (6–10 g). Our findings suggested that PMMA spacers loaded with relatively lower vancomycin concentrations (1–4 g) did not interfere with new bone formation, whereas spacers loaded with relatively higher vancomycin concentrations (6–10 g) had negative effects on bone formation.

## Introduction

A critical-sized bone defect frequently occurs in cases of high-energy trauma, osteomyelitis, or tumour resection. Despite multiple approaches having been described, including autogenous cancellous bone graft, vascular bone transplantation, Iliazarov distraction osteogenesis, the Masquelet technique, and even amputation, the reconstruction of a critical-sized defect is one of the greatest challenges in the orthopaedic field^1^. The Masquelet technique, first described by Alain Masquelet in the 1980s, is a relatively new, two-stage procedure that allows for the reconstruction of segmental bone defects of up to 25 cm^2,3^. The first stage consists of thorough debridement, temporary implantation of polymethyl methacrylate (PMMA), and the stability of defects, which is conducive to the formation of an induced membrane^4^. In the second stage, the induced membrane is opened for PMMA removal, and an adequate volume of graft material is implanted into the bone defect^5^. The autologous induced membrane formed surrounding the PMMA spacer during the first phase of the Masquelet technique is critical to the bone consolidation of the bone graft in the second phase^6,7^. Although some previous studies have focused on the osteogenesis mechanism of the Masquelet technique, the exact regulatory mechanisms of bone defect repair remain unclear^8–11^.

Whether antibiotics should be included remains greatly debated^12,13^. Many studies have recommended the use of antibiotic-loaded PMMA cement during the first stage of Masquelet^14–16^. Additionally, a systematic review and a meta-analysis have also suggested that the antibiotic-loaded PMMA cement might prevent repeated surgeries for bone grafting^17^. Previous studies have proved that the inclusion of antibiotics in the PMMA spacer can alter the characteristics of the induced membrane^18^, and that antibiotics play an important role in the gene expression profile of induced membrane and the regeneration of infected defects^19^. To the best of our knowledge, no study has focused on the effect of antibiotics on bone regeneration in the second phase of Masquelet. Vancomycin is one of the most commonly used antibiotics in Masquelet, and there remains debate over what dose of antibiotics should be used^20^. Furthermore, the dose of vancomycin applied in previous studies has been varied from 1 to 10 g, and there has been no consensus yet on a recommended dosage in previous clinical practice^20,21^. Our previous study has evaluated the effects of different vancomycin on the formation of the induced membrane in rabbit models of Masquelet technique, which revealing that PMMA spacers loaded with relatively low concentrations of vancomycin (1–4 g per cement dose) did not interfere with the proliferation, osteogenesis, and angiogenesis activity of induced membranes, and even promoted their activity^22^. Whereas no study has focused on the effect of different vancomycin concentrations on bone defect repair.

The present study intended to construct a new Masquelet model (PMMA spacer loaded with varying vancomycin concentrations). The main objective of this study was to determine the effect of a PMMA spacer loaded with different vancomycin concentrations on bone defect repair. Bone regeneration was assessed by X-ray and micro-CT analysis.

## Materials and methods

### Spacer preparation and animal grouping

Spacers consisting of PMMA (per 40 g kit, Heraeus Medical GmbH, Wehrheim, Germany) and varying vancomycin concentrations (0, 1, 2, 4, 6, 8, and 10 g, Zhengde Pharmaceutical Company, Taiwan, China) were prepared. As specified in our previous research^22^, following mixing of the vancomycin, bone cement powder, and the monomer liquid to achieve a dough phase, the mixture was poured into a prefabricated mould to form the hollow cylindrical spacer (height 10 mm, external diameter 3 mm, and internal diameter 0.8 mm). An entire 40 g kit was used for each vancomycin concentrations, and these spacers were stored in an aseptic container for later use.

All experiments were approved by the Medical Ethics Committee of Xiangya hospital Central South University (No. 202103710), and experiments were performed in accordance with relevant guidelines and regulations. And this study was reported in accordance with ARRIVE guidelines. Twenty-eight New Zealand white rabbits, weighing approximately 2000–2500 g and aged 3 months, were evenly randomised into seven groups (n = 4), which were used for the generation of the Masquelet model. One additional rabbit was sacrificed to harvest the allogeneic iliac bone during the second phase of Masquelet. In addition, another four rabbits served as blank controls, which received no PMMA implantation or bone graft.

### Surgical procedure of the Masquelet technique

Following intramuscular anaesthesia (xylazine hydrochloride, 20 mg/kg body weight), a critical bone defect (10 mm) was created at approximately the middle of the left radial shaft with a high-speed power drill. The cylindrical PMMA spacer was placed in the defect, and this was subsequently fixed intramedullary with a retrograde Kirschner’s wire (diameter 0.8 mm). The entry point of the Kirschner’s wire was at the distal radius. After penetrating the medullary cavity and the PMMA spacer, the tip of the Kirschner’s wire was drilled into the proximal radius. Additionally, the tail of the Kirschner’s wire was curved for the removal of the PMMA spacer in the second phase of Masquelet. Immediately and 2 and 4 weeks after the operation, X-ray imaging under anesthesia was performed to evaluate fixation stability.

**Figure 1:**
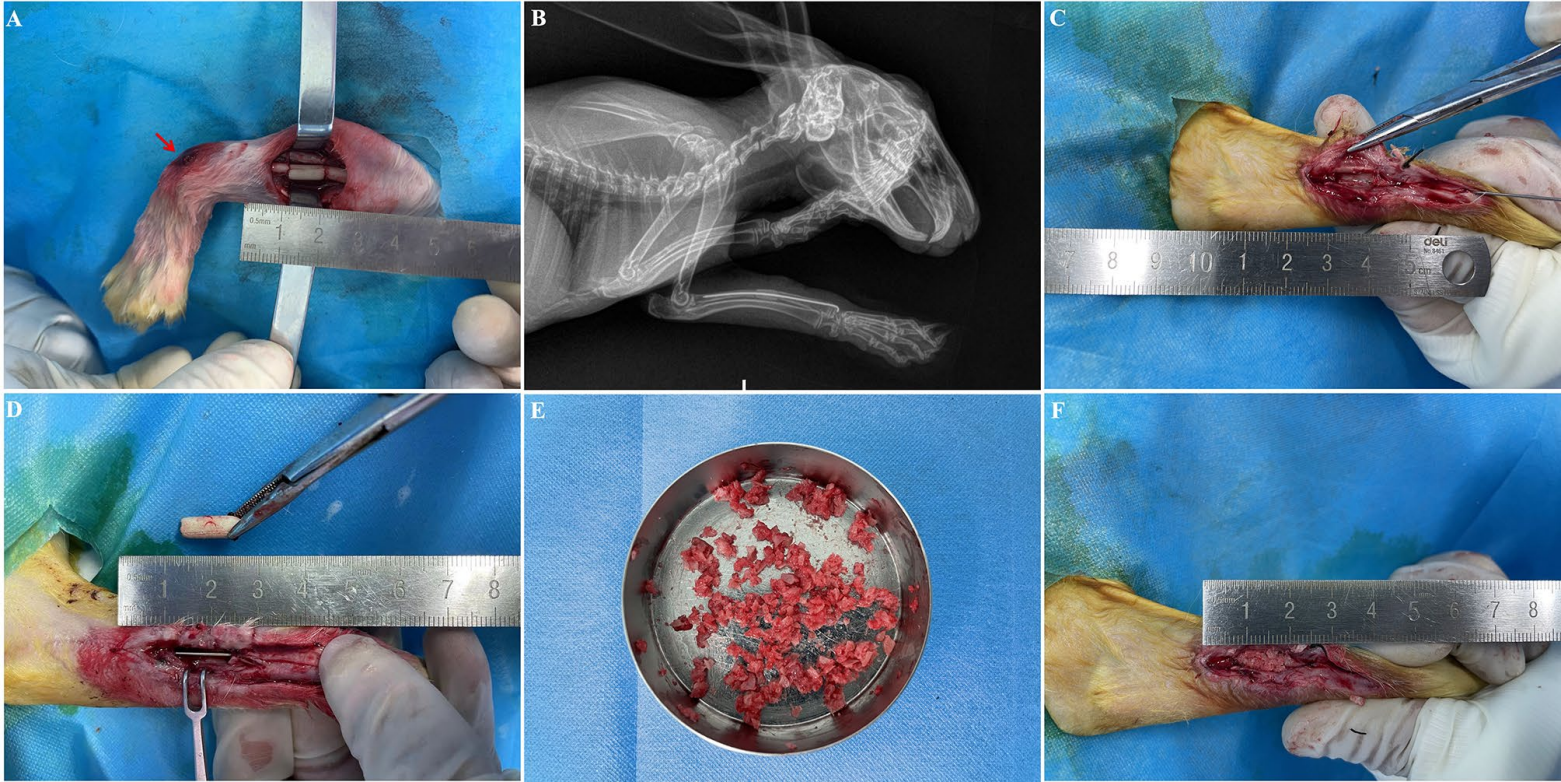
Application of the Masquelet technique. A critical bone defect (10 mm) was created at approximately the middle of the left radial shaft, and the defect was inserted with a cylindrical PMMA spacer and subsequently fixed intramedullary with a retrograde Kirschner’s wire; the red arrow indicated the internal fixation point, and the Kirschner’s wire was curved by a clamp at this puncture spot by a clamp **(A)**. Postoperative X-ray revealed the good location and rigid internal fixation of the PMMA spacer **(B)**. Four weeks after PMMA implantation, the pre-curved tail of the Kirschner’s wire was exposed and pulled out partly for the subsequent removal of the PMMA **(C)**. The Kirschner’s wire was reinserted into the cavity of the radius **(D)**. Allogeneic iliac bone was harvested and cut into pieces for further bone grafting **(E)**. An adequate volume of graft material was implanted into the bone defect **(F)**.

Four weeks after PMMA implantation, the rabbits were anaesthetised and the induced membrane was opened longitudinally. The pre-curved tail of the Kirschner’s wire was exposed and pulled out partly for the subsequent removal of the PMMA. After skin preservation and sterilization, allogeneic iliac bone was harvested from one non-operated rabbit, and these bone blocks were cut into pieces (diameter 1–2 mm) and then kept in a covered container with additional moisture for later grafting. The average operation time of grafting was nearly ten minutes, so the process of bone grafting in all rabbits was performed on the same day with the same iliac bone. Subsequently, the Kirschner’s wire was reinserted into the cavity of the radius and an adequate volume of graft material was implanted into the bone defect. Then, the induced membrane was tight sutured with 4–0 absorbable sutures to create a contained space for the graft. Finally, the fascia and skin were closed with 4–0 absorbable sutures. The bone defect of the blank control group was established, which was also fixed with Kirschner’s wire, but received no bone graft (Fig. 1).

### X-ray analysis

Immediately and 2, 4, 6, and 8 weeks post-operatively, the rabbits were anaesthetised and X-ray (drVET1600, Perlove, China) was applied to observe the repair of bone defects, which was quantitatively scored using the Lane-Sandhu scoring system by one independent observer blinded to the grouping^23^.

### Micro-CT analysis

Eight weeks post-operatively, the rabbits were euthanized for further micro-CT analysis. The left forelimbs were amputated and the Kirschner’s wires were removed, and then the samples were fixed with paraformaldehyde prior to micro-CT scanning and between scanning and histological processing. The amount of bone formation in the second phase was determined using a micro-CT scanner (Skyscan 1076, Kon-Tich, Belgium), with a voxel resolution of 20 μm, 70 kV energy setting, and an intensity of 130 μA. The three-dimensional structures of the bone defects were reconstructed using the Data Viewer (version 1.5.6.2) and Mimics Research (version 20.0) analysis software. The 3D reconstruction was used to blindly qualify each sample’s bridging as either united, unclear (obvious void) or empty, by one independent observer blinded to the grouping^24,25^. The bone volume (BV), total volume (TV), BV/TV, trabecular number (Tb.N), and trabecular thickness (Tb.Th) were analysed using CTAn (version 1.11) software.

### Histological analysis

After micro-CT scanning, the left forelimbs were decalcified in 10% Ethylene Diamine Tetraacetic Acid and dehydrated in graded alcohol. The haematoxylin/eosin (H&E) and Masson’s Trichrome staining were performed to evaluate new bone formation of the defects. And immunofluorescence (IF) staining was performed to detect the level of OCN expressions in the defects. A primary antibody of anti-OCN (Proteintech, USA) and a secondary antibody of conjugated goat anti-rabbit IgG(H + L) (Proteintech, USA) were used in the staining process. And these sections were observed by Fluorescence microscope (Nikon, Tokyo, Japan).

### Statistical analysis

All data are presented as means ± standard deviations. Differences among groups were calculated by one-way ANOVA using SPSS software. Additionally, LSD or Dunnett’s T3 test were used for multiple comparisons. Differences were considered as significant at P ≤ 0.05.

### Ethics approval

The medical ethics committee of Xiangya Hospital Central South University gave ethical approval of this research. (No. 202103710).

### Consent to participate

All authors are fully involved in the study and preparation of the manuscript.

### Consent to publish

The authors claim that none of the material in the paper has been published or is under consideration for publication elsewhere, and that if accepted, will not be published elsewhere in the same form, in English or in any language, without the written consent of the publisher.

## Results

**Figure 2:**
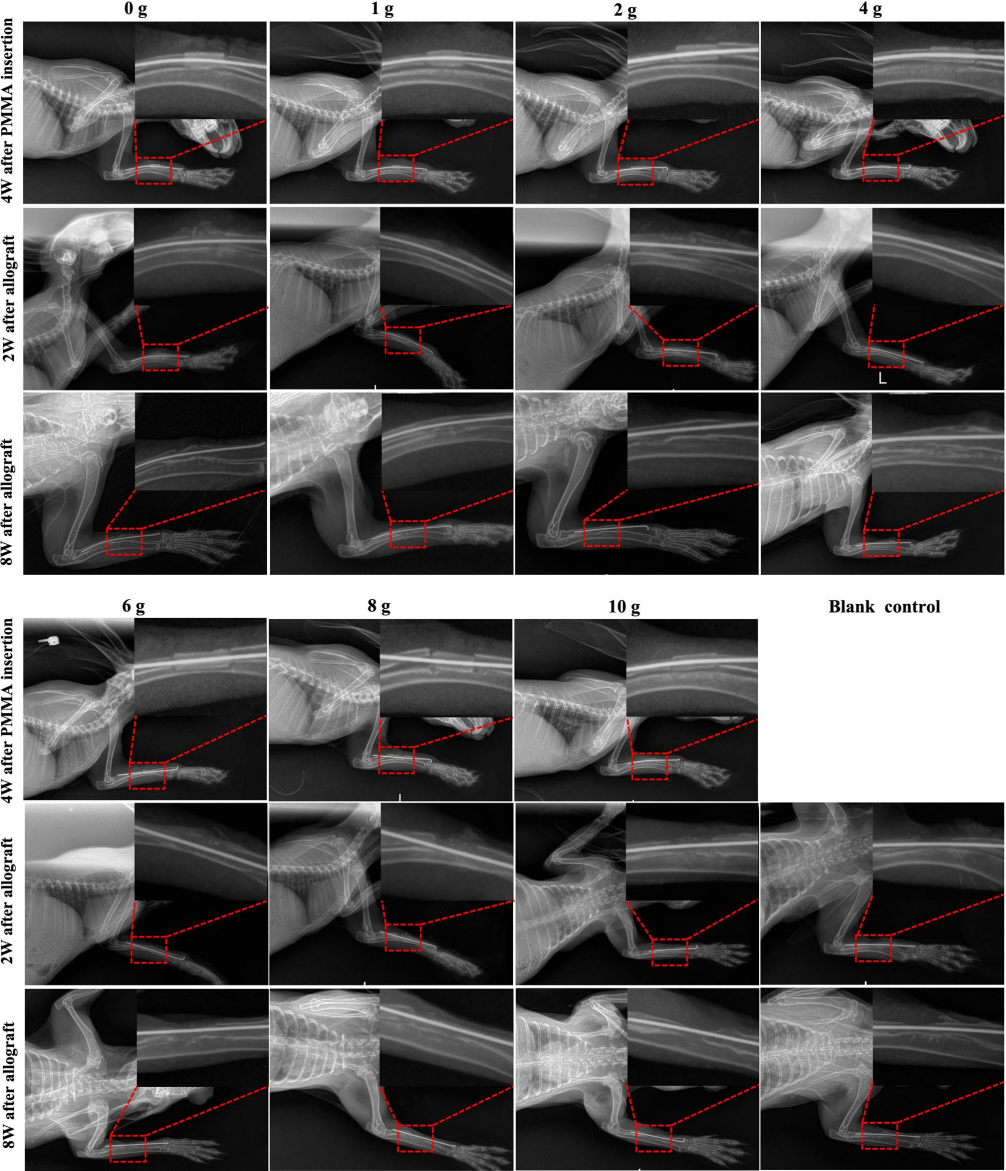
Representative X-ray analysis. The bone defect of the blank control group was fixed with Kirschner’s wire, but received no bone graft.

**Figure 3:**
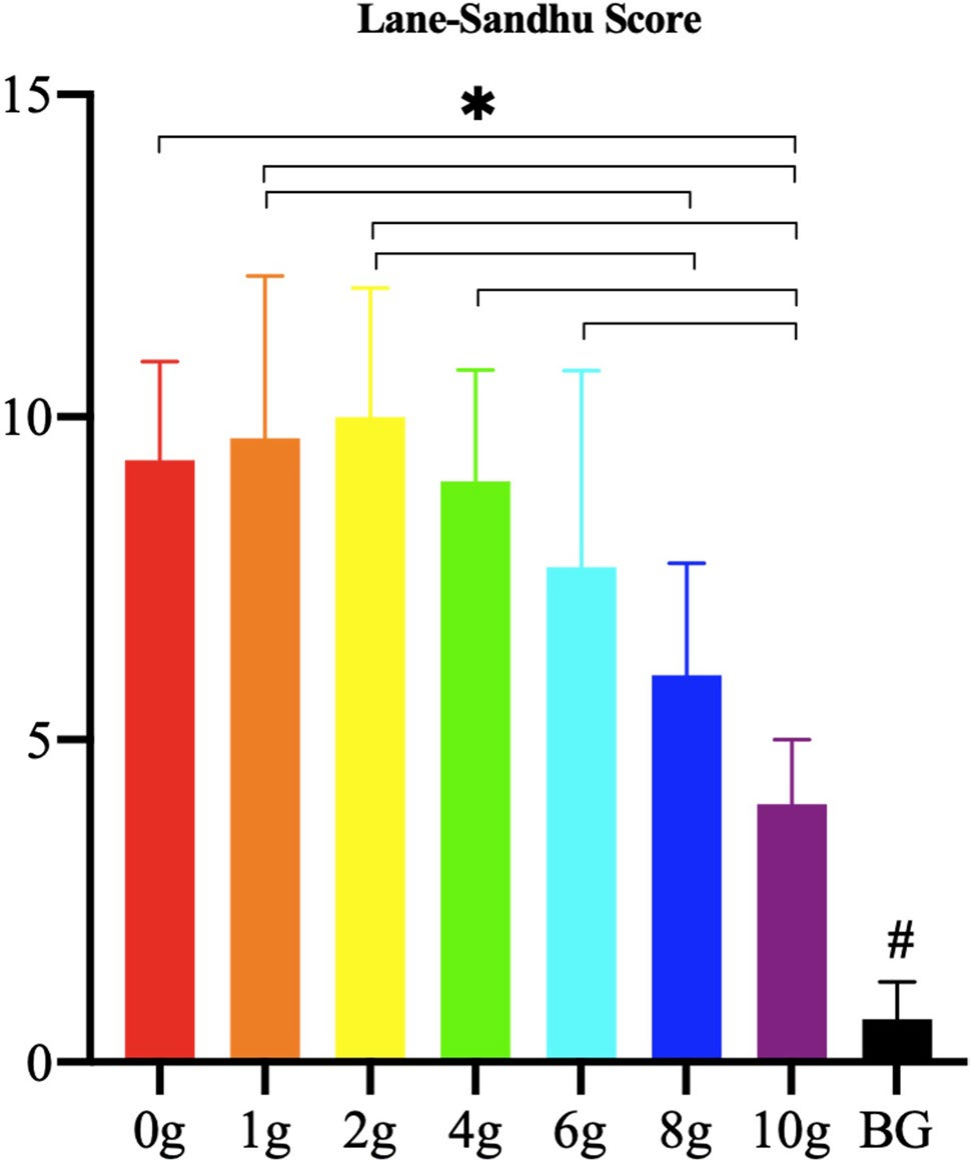
The quantitative analysis of Lane-Sandhu Score using X-ray. (*: *P* < 0.05; **#**: *P* < 0.05, compared with the groups with PMMA implantation; *BG* blank control group).

Representative X-ray characteristics are presented in Fig. 2. The spacer and bone defects were stable at the fourth week after PMMA implantation. Following bone grafting, the residual allograft and some new bone formation were observed at the second week after the allogeneic iliac bone graft, and more cortical bone was formed to bridge the gap, and the bone marrow cavity was formed at the eighth week after the graft, particularly in the groups with a relatively lower vancomycin concentration (0–4 g). There was little to no new bone formation in the defects of the blank control group. Additionally, there was only limited new bone formation when the concentration of vancomycin was 6 g or above. The quantitative analysis of X-ray in the eighth week after the operation revealed that the radiological score in the blank control group was significantly decreased compared with that in the groups with PMMA and vancomycin. The score in the group with 10 g vancomycin was significantly decreased compared with that in the groups with 0–8 g vancomycin, the score in the group with 8 g vancomycin was significantly decreased compared with that in the groups with 1 and 2 g vancomycin, and there was no significant difference among the other groups (Fig. 3).

**Figure 4:**
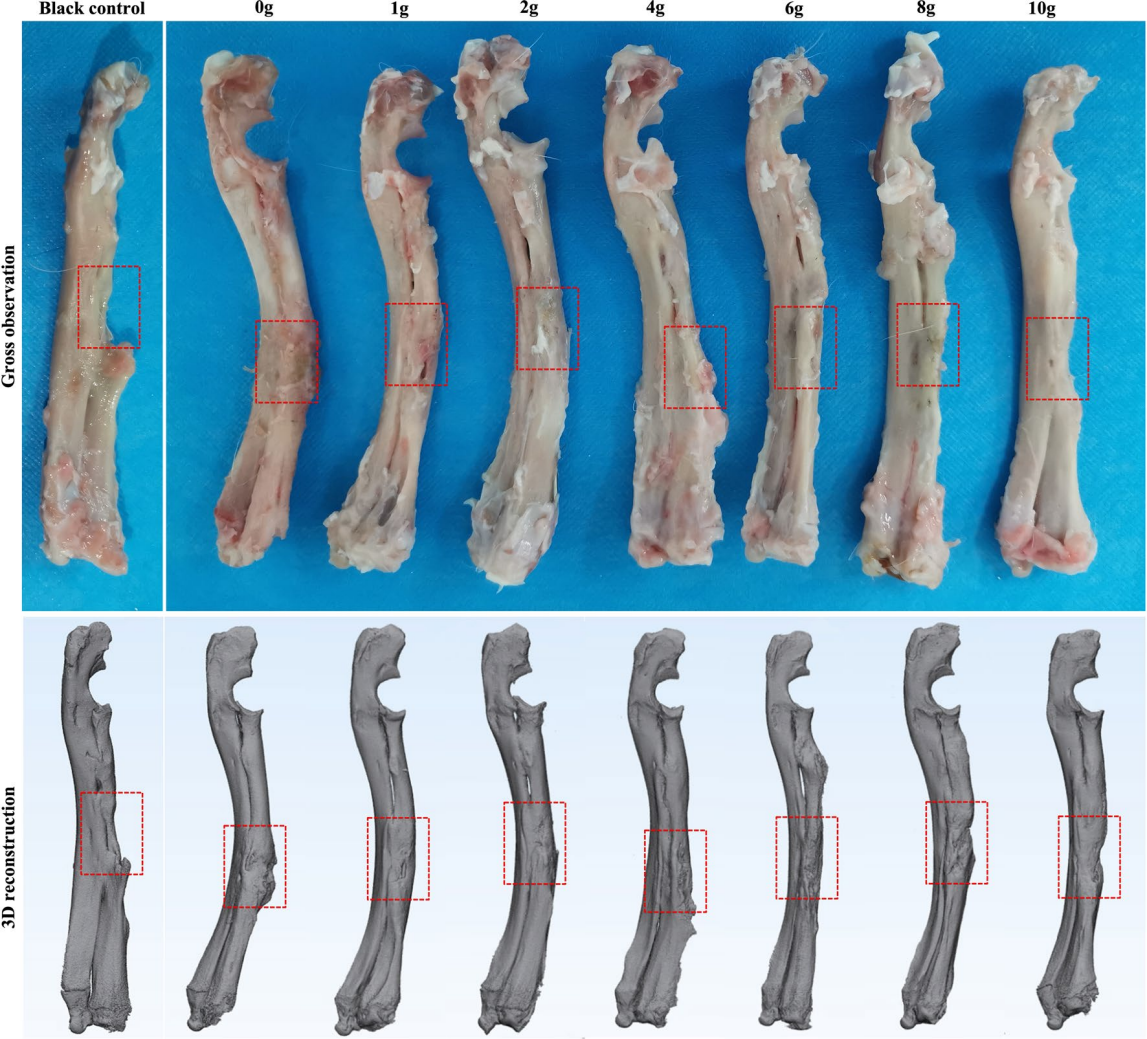
Representative gross observation and 3D reconstruction of the left forelimbs.

**Figure 5:**
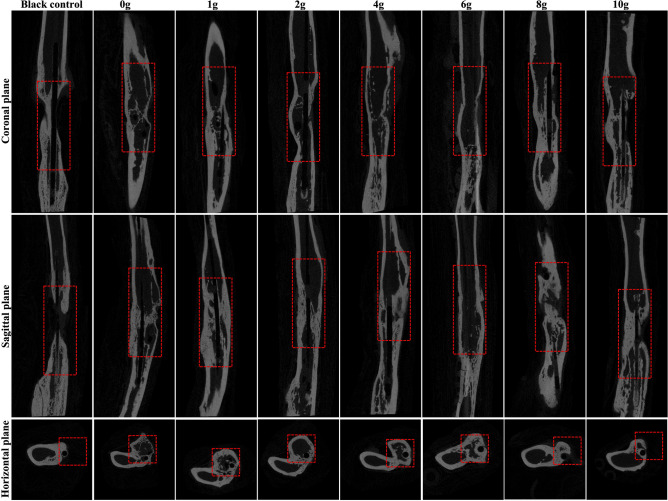
Representative micro-CT results in the coronal, sagittal, and horizontal planes.

Representative gross observation and three-dimensional reconstruction of the left forelimbs are presented in Fig. 4. And the micro-CT examinations in the coronal, sagittal, and horizontal planes were shown in Fig. 5. These results revealed that clear new bone was formed around the bone defects in the groups with a relatively low vancomycin concentration (0–4 g). Whereas there was very limited bone formation in the groups with relatively high vancomycin (6–10 g), and this was less than in the blank control group. Quantitatively, more samples in the groups receiving 0–4 g vancomycin were united than samples in the groups receiving 6–10 g vancomycin (n = 14 of 16, and 4 of 12, respectively, *p* < 0.05). And there was either an obvious disconnected or clear continuous void of the radial shaft in the rest samples receiving 0–4 g vancomycin (n = 2 of 16) and in the rest samples receiving 6–10 g vancomycin (n = 8 of 12). Additionally, there was an obvious disconnected in the blank control group (n = 4 of 4).

**Figure 6:**
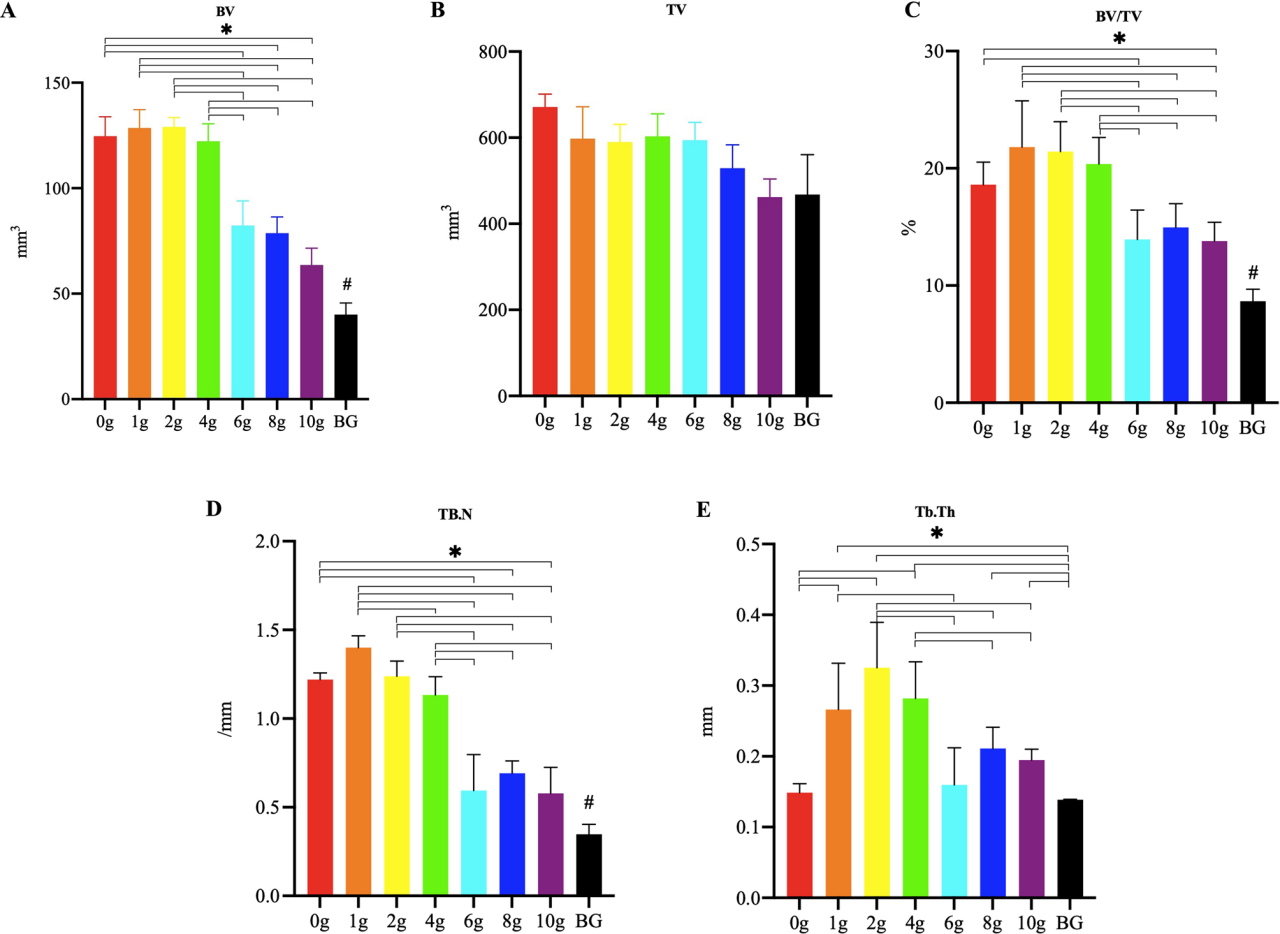
The quantitative analysis of BV, TV, BV/TV, Tb. N, and Tb. Th using micro-CT. (*: *P* < 0.05; **#**: *P* < 0.05, compared with the groups with PMMA implantation; *BG* blank control group).

The quantitative analysis of the micro-CT is presented in Fig. 6. The BV in the blank control group was significantly decreased compared with that in the groups with PMMA and vancomycin, and the BV in the groups with 6, 8, and 10 g vancomycin was significantly decreased compared with that in the groups with 0, 1, 2, and 4 g vancomycin. The results of TV showed that there was no significant difference among the groups. The BV/TV in the blank control group was significantly decreased compared with that in the groups with PMMA and vancomycin, the BV/TV in the groups with 6 and 10 g vancomycin was significantly decreased compared with that in the groups with 0, 1, 2, and 4 g vancomycin, and the BV/TV in the group with 8 g vancomycin was significantly decreased compared with that in groups with 1, 2, and 4 g vancomycin. TB.N in the blank control group was significantly decreased compared with that in the groups with PMMA and vancomycin. TB.N in the groups with 6, 8, and 10 g vancomycin was significantly decreased compared with that in the groups with 0, 1, 2, and 4 g vancomycin, and the TB.N in the group with 4 g vancomycin was significantly decreased compared with that in the group with 1 g vancomycin. Tb.Th in the blank control group was significantly decreased compared with that in groups with 1, 2, 4, 8, and 10 g vancomycin. Tb.Th in the groups with 8 and 10 g vancomycin was significantly decreased compared with that in the groups with 2 and 4 g vancomycin, and Tb.Th in the group with 6 g vancomycin was significantly decreased compared with that in the groups with 1 and 2 g vancomycin, and Tb.Th in the group with 0 g vancomycin was significantly decreased compared with that in the groups with 1, 2, and 4 g vancomycin. There was no significant difference among the other groups.

**Figure 7:**
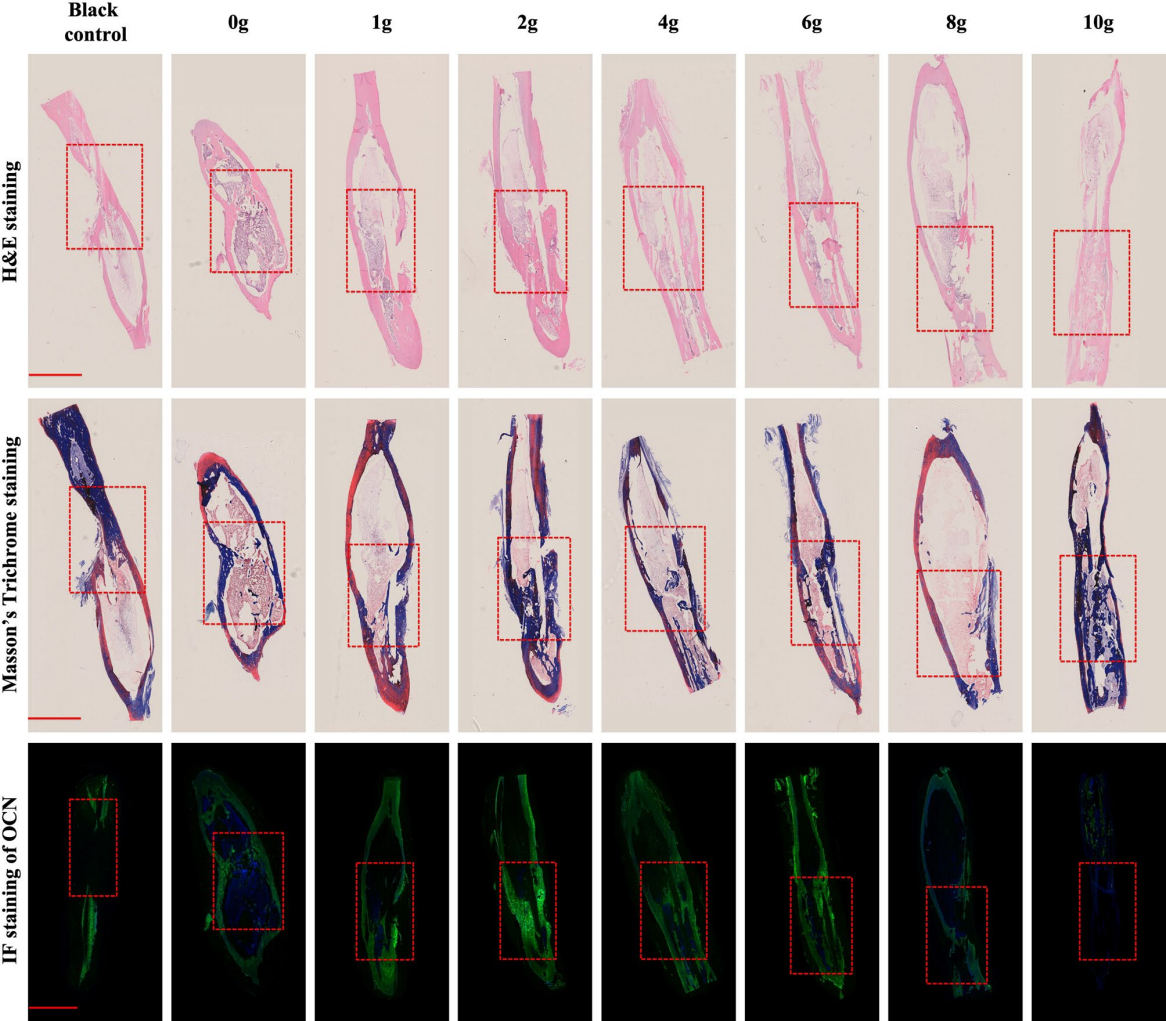
Representative histological sections of bone defects with H&E staining, Masson’s Trichrome staining, and IF staing of OCN. The red dotted box indicated areas of bone defects (scale bar: 1 mm).

Representative histological analyses of bone defects by H&E, Masson’s Trichrome and IF staining are presented in Fig. 7. As the results show, red collagenous fibers in H&E staining and blue fibrous tissue in Masson’s Trichrome staining was observed, which revealing new bone formation in all groups. And obvious bone cortex and medullary cavity were formed in groups with relatively low vancomycin concentration (0–4 g), whereas there was just little bone formation in the blank control group and in the groups with relatively high vancomycin (8–10 g). And as shown in Fig. 7, the qualitative results of IF staining indicated obvious expression of OCN in groups with relatively low vancomycin concentration (0–6 g), whereas there was just little protein expression of OCN in the blank control group and in the groups with 8, and 10 g vancomycin.

## Discussion

The influence of antibiotics on the formation of induced membrane has been subject to debate, and the clinical use of antibiotics varies greatly from one surgeon to another^26,27^. The dose of vancomycin (one of the most commonly used antibiotics in the Masquelet technique) varied from 1 to 10 g in previous clinical practice, and there has been limited evidence in vitro to suggest that mesenchymal stem cells and osteoblasts could be affected by varying types of antibiotics^21,28,29^. Additionally, the quality of the induced membrane could also be significantly influenced by antibiotics^18,19^. The effect of vancomycin on the quality of defect repair remains uncertain. The main objective of our study was to determine the effect of a PMMA spacer loaded with different vancomycin concentrations on bone defect repair.

A critical-sized bone defect is defined as a defect with a minimum length that cannot heal spontaneously, which is normally more than 1.5 to 2 times the diameter of the bone diaphysis^30^. In the present study, a 10-mm-length bone defect was created in all groups. The results showed that rabbits in the blank control group exhibited no bone healing, in contrast to the defects treated by the Masquelet technique, revealing that a critical-sized bone defect was created. Following PMMA implantation, appropriate fixation of the bone defect and PMMA spacer should be achieved. Typically, bone defects are fixed with plates and screw system. In our research, the medullary cavity and the PMMA spacer were penetrated with a retrograde Kirschner’s wire. The X-ray results of our research showed that reliable stability of the bone defect was achieved. The retrograde Kirschner’s wire could minimize the interference of fixation materials on formation of the induced membrane, and might also lead to the lack of stabilization in the defects. And there was an obvious disconnected in the blank control group. The bone nonunion in the blank control group was partly attributed to the lack of stabilization in the defects.

The X-ray, micro-CT and histological results of the present study showed that greater bone formation was observed in the Masquelet groups compared with the blank group, revealing that the new Masquelet model (PMMA spacer loaded with varying vancomycin concentrations) could effectively promote the repair of bone defects. These results further validated that the rabbit model of the Masquelet technique was successfully established. Additionally, the new bone formation in the groups with lower vancomycin was relatively higher than that in the group without vancomycin, although the difference was not always statistically significant. These results suggested that local administration of low-dose vancomycin in a PMMA spacer did not inhibit bone formation. Previous research has also highlighted that local antibiotic delivery plays an important role in bone repair in addition to the antimicrobial properties^19,31^. Generally, more new bone formation was observed in the groups with a lower vancomycin concentration (1, 2, 4 g) compared with groups with higher vancomycin (6, 8, 10 g), which revealed that the process of bone formation was restricted by high-dose vancomycin. These results were in agreement with previous in vitro studies, whereby high-dose vancomycin could impair the viability and osteogenic differentiation of mesenchymal stem cells^32,33^.

Exploring the influencing of varying vancomycin concentrations on bone regeneration could help to further improve the clinical effects of Masquelet technique. Many researches have suggested that antibiotics play an important role in the gene expression profile of induced membrane^18,19^. Our previous study has revealed that the addition of vancomycin in PMMA spacer does not impair the osteogenesis of induced membranes at a relatively low concentration, and that spacer loaded with relatively high concentrations of vancomycin has negative effects on osteoblast viability^22^. The changes of induced membrane might further affect the activity of bone formation in the second stage of Masquelet. Still the exact mechanism of vancomycin on the quality of defect repair remains uncertain. Further researches should be performed to explore the exact mechanism of varying vancomycin concentrations on bone regeneration in Masquelet technique.

There have been some limitations in our research. First, relatively low sample number (n = 4) has been used and relatively short duration (8 weeks) of defect repair has been examined. While statistically significant results of the main quantitative outcome were achieved. And a period of 8 weeks in the second stage of Masquelet technique has been chosen since that massive new bone could be formed over a period of 8 weeks in rabbit model, and many previous studies have also revealed that a short period of 6–8 weeks in rabbits model is appropriate for the evaluation of new bone formation^34,35^. Still, different results might have been obtained in a longer time span. Second, vancomycin is only one of the typical antibiotics used in Masquelet technique, and gentamycin, clindamycin, and tobramycin are also commonly used in PMMA spacer. It is unclear whether similar results would be obtained with different antibiotics. Finally, although there have been remarkable similarities between animals and human research in Masquelet technique^11,36^, further experiments and clinical studies are also necessary to verify our results.

## Conclusion

PMMA spacers loaded with relatively low vancomycin concentrations (1–4 g) did not interfere with new bone formation, whereas spacers loaded with relatively high vancomycin concentrations (6–10 g) had negative effects on bone formation.
